# Corrosion Resistance Enhancement of CoCrFeMnNi High-Entropy Alloy with WC Particle Reinforcements via Laser Melting Deposition

**DOI:** 10.3390/ma16134701

**Published:** 2023-06-29

**Authors:** Zhen Peng, Zize Fan, Muhammad Raies Abdullah, Congcong Ren, Jinfeng Li, Pan Gong

**Affiliations:** 1 School of Materials Science and Engineering, Jiangsu University, Zhenjiang 212013, China; 2 Institute of Materials, China Academy of Engineering Physics, Mianyang 621907, China; lijinfeng305@126.com; 3 Research Institute of Huazhong University of Science and Technology in Shenzhen, Shenzhen 518057, China; 4 State Key Laboratory of Materials Processing and Die & Mould Technology, School of Materials Science and Engineering, Huazhong University of Science and Technology, No. 1037 Luoyu Road, Wuhan 430074, China

**Keywords:** high-entropy alloys, corrosion behavior, laser melting deposition, the passivation film, the potentiodynamic polarization curves

## Abstract

In the present work, a WC particle-reinforced CoCrFeMnNi high-entropy alloy (HEA) was fabricated by laser melting deposition (LMDed). The LMDed CoCrFeMnNi high-entropy alloy (CoCrFeMnNi) composite is primarily comprised of a face-centered cubic (FCC) crystal structure. However, in the case of CoCrFeMnNi with 2.5 wt.% WC, it exhibits a combination of an FCC matrix and a ceramic phase known as M_23_C_6_. The corrosion behavior of CoCrFeMnNi and CoCrFeMnNi with 2.5 wt.% WC particle in 0.5 M H_2_SO_4_ was comparatively investigated. Compared with CoCrFeMnNi, the passive film formed on the CoCrFeMnNi with 2.5 wt.% WC had a more stable and stronger protective property. The corrosion current density of the CoCrFeMnNi with 2.5 wt.% WC dropped by 149.1% compared to that of the CoCrFeMnNi, indicating that the CoCrFeMnNi with 2.5 wt.% WC had better corrosion resistance than that of the CoCrFeMnNi.

## 1. Introduction

The development of high-entropy alloys (HEAs) has drawn significant interest since the pioneering work in 2004 by Yeh et al. and Cantor et al. [1,2]. In contrast to the conventional method with only one dominant element in the alloy, HEAs are composed of multiple elements with near-equal atomic percentages. Due to the “four core effects” defined by Yeh, HEAs can exhibit remarkable mechanical and functional properties, such as excellent thermal stability, wear, and oxidation resistance corrosion resistance [3,4,5,6,7,8,9,10].

Traditional manufacturing processes, such as vacuum arc melting, mechanical alloying, and powder metallurgy, have been extensively used to fabricate HEAs [11,12,13,14,15]. However, these HEAs usually have restricted shapes and coarse grains, which limits the wide application of HEAs. In recent years, several studies on additively manufactured (AM) HEAs have been carried out [16,17,18,19,20]. Additive manufacturing technology offers a rapid and efficient approach to fabricate alloys with gradient or complex shapes, making it a valuable tool in advancing the development of high-entropy alloys. This technique possesses several advantageous features, including unrestricted forming size and structure, free-forming capabilities, net shaping, and precise manufacturing. In contrast to conventional preparation methods, AM provides better control over structural uniformity and enables the production of ultra-fine grains, leading to enhanced overall mechanical properties of WC-containing HEAs. As a result, AM holds significant potential in ensuring the structural integrity and enhancing the comprehensive mechanical performance of WC-containing HEAs, contributing to their advancement and application. As one of the most widely used AM methods, laser melting deposition (LMD) can fabricate metal parts in complex shapes with high precision and excellent performance [21,22,23,24,25,26].

The CoCrFeMnNi high-entropy alloy is a well-researched material known for its outstanding mechanical properties, particularly at cryogenic temperatures. This is attributed to the activation of diverse deformation mechanisms, including dislocation and twin-mediated processes, within its single-face FCC structure. Notably, studies have revealed that additive manufacturing techniques can further enhance the CoCrFeMnNi HEA by producing finer grain sizes and increasing its overall strength, surpassing the properties of conventionally manufactured counterparts. In order to advance the comprehensive properties of high-entropy alloys, there is a continual need for novel modification methods. In recent times, the incorporation of ceramic particles such as carbides and nitrides into HEAs have emerged as a promising approach to enhance their properties. This strategy enables the customization of structures and facilitates the synergistic combination of mechanical and chemical attributes in HEAs. By incorporating ceramic particles, researchers aim to achieve optimized performance and further unlock the potential of these materials [4,27,28,29,30,31,32].

In this study, a CoCrFeMnNi high-entropy alloy with 2.5 wt.% WC particles was fabricated using laser melting deposition. A comparative analysis was conducted to examine the electrochemical corrosion behavior of the CoCrFeMnNi and CoCrFeMnNi with 2.5 wt.% WC particles in a 0.5 M H_2_SO_4_ environment. The aim was to gain insights into the corrosion resistance enhancement. The investigation included microstructural characterization and examination of the composition uniformity of the alloy. By comprehending these factors, the underlying reasons behind the improved corrosion resistance could be identified in this work.

## 2. Materials and Methods

### 2.1. Sample Preparation

Laser melting deposition (LMD) was utilized to fabricate the CoCrFeMnNi and CoCrFeMnNi with 2.5 wt.% WC high-entropy alloy particles. The average particle sizes of pre-alloyed CrMnFeCoNi powder and WC powder were analyzed by Microtrac S3500 (Microtrac, Largo, FL, USA) laser particle size analyzer, which were approximately 120 μm and 10 μm, respectively. The two powders are mixed according to the designed proportion. The mixed powder is heated to 80 °C and dried for 2 h and then cooled to room temperature in a vacuum chamber before use. The mixed powder is transported to the laser molten pool through a closed loop powder supply unit, and deposited continuously on 316 L stainless steel substrate by a reciprocating multi-layer scanning under 1000 W laser power and 500 mm/min scanning speed. During deposition, the atmosphere was under the protection of argon, and the oxygen content in the room was below 20 ppm. The mixed powder is delivered to the chamber through the coaxial nozzle at an argon flow rate of 15–18 L/min and a feed rate of 7–9 g/min. The width of a single deposition track was approximately 35 mm, the thickness was approximately 4 mm. After each layer was deposited, the laser head would rise to a certain height until the deposition height reaches 40 mm.

### 2.2. Microstructural Characterization

Electric spark corrosion was used to cut thin-walled samples with at least 4 mm away from the substrate. Samples were polished with sandpaper from 200 #, 400 #, 600 #, to 3000 #, followed by mechanical polishing until there were no obvious scratches under a 400× optical microscope. X-ray diffraction (XRD) analysis of the particles’ crystalline structure was conducted using a Japan Nigaku D/max-RB X-ray diffraction spectrometer equipped with Cu-Kα radiation. The scanning angle for the analysis ranged from 15° to 90°. The size and microstructure of samples are characterized by S-4800 SEM and JEM 200CX transmission electron microscope (TEM) with 200 kV operating voltage. Aqua regia was used for corrosion before SEM testing. For TEM testing, the samples were grinded to below 100 µm, and then punched a hole of *Φ*3 mm. The ion thinning method was used to thin them until met the requirements of TEM testing. JEM 200CX transmission electron microscope (TEM) with 200 kV operating voltage. The composition of the etched passivation film was characterized using FEI EscaLab 250Xi X-ray photoelectron spectroscopy (XPS).

### 2.3. Electrochemical Measurements

The electrochemical workstation was used to characterize the corrosion resistance of the alloy. The corrosion medium was 0.5 M H_2_SO_4_ solution. The test surface size of 10 mm × 10 mm, the back side of the test surface is connected to the copper wire using conductive adhesive, and the other surfaces are wrapped and sealed with epoxy resin to avoid contacting with the test solution. During the test, a three-electrode system is selected, with the platinum plate electrode as the auxiliary electrode. The connecting wire of the sample is encapsulated with rubber resin, and the surface exposed for the test is used as the working electrode. The potentiodynamic polarization curve and AC impedance curve of the alloy was obtained. The scanning rate was 3 mv/s during the action potential polarization test. When conducting the AC impedance test, the test frequency range is 10^−2^–10^6^ Hz, and the amplitude is 5 mv.

## 3. Results and Discussion

### 3.1. Microstructure before Corrosion

**Figure 1:**
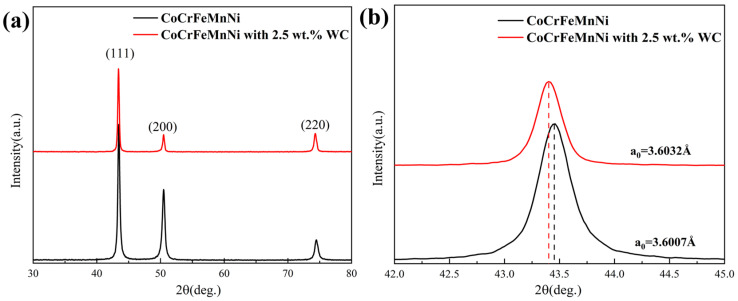
(**a**) X-ray diffraction patterns of CoCrFeMnNi and CoCrFeMnNi with 2.5 wt.% WC samples, (**b**) partial enlargement of the XRD pattern in (**a**).

Figure 1a illustrates the X-ray diffraction (XRD) analysis results of the LMDed fabricated CoCrFeMnNi and CoCrFeMnNi with 2.5 wt.% WC high-entropy alloy samples. The two alloys show a single-phase FCC structure, and the peak of WC is not found in the XRD results of CoCrFeMnNi with 2.5 wt.% WC samples. Figure 1b is an enlarged view of the (111) peak. It can be seen from the figure that the main peak of the CoCrFeMnNi with 2.5 wt.% WC sample shifted significantly to the left side. According to the Bragg equation (2dsin θ = λ), the observed difference in lattice constants between the LMDed CoCrFeMnNi sample with 2.5 wt.% WC (a_0_ = 3.6032 Å) and the CoCrFeMnNi sample (a_0_ = 3.6007 Å) can be attributed to the incorporation of WC particles into the HEA matrix. The addition of WC can introduce lattice strain and result in a slight expansion of the lattice. The presence of WC particles may cause lattice distortion and contribute to the observed increase in lattice constant. It is worth noting that the difference in lattice constant may also be influenced by factors such as processing conditions, composition variations, and the distribution of WC particles within the HEA matrix [22].

**Figure 2:**
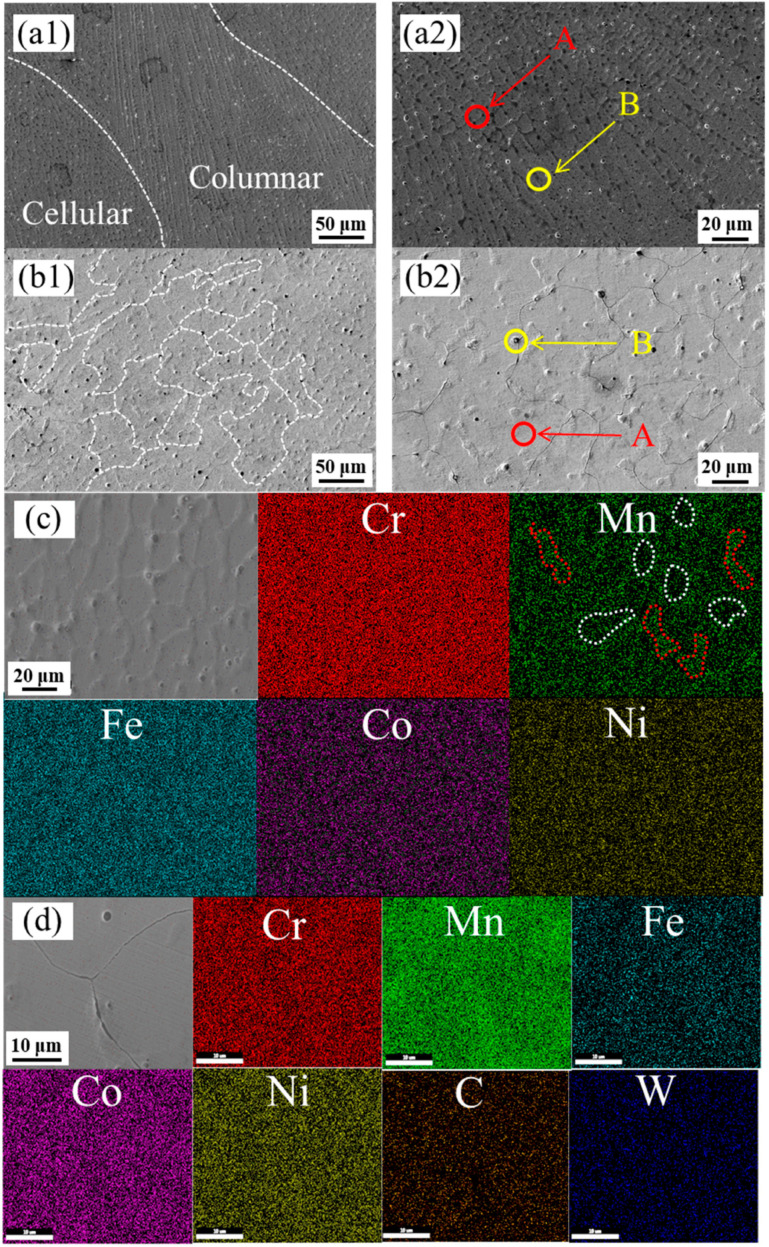
Microstructural characterization of samples (**a1**) SEM of CoCrFeMnNi, (**a2**) partial enlargement view of a1, (**b1**) SEM of CoCrFeMnNi with 2.5 wt.% WC, (**b2**) partial enlargement view of b1 (**c**) EDS mapping of CoCrFeMnNi, (**d**) EDS mapping of CoCrFeMnNi with 2.5 wt.% WC.

Figure 2 shows the SEM images and EDS mapping of CoCrFeMnNi and CoCrFeMnNi with 2.5 wt.% WC. It can be seen from the SEM image that is a mixed structure of dendritic and cytosolic crystals, while CoCrFeMnNi with 2.5 wt.% WC are mostly columnar crystals. Compared with CoCrFeMnNi, CoCrFeMnNi with 2.5 wt.% WC shows a finer grain size. As can be seen from the EDS mapping in Figure 2c, Mn and Fe elements in the CoCrFeMnNi are separated, which is consistent with the results of Cantor and Salishchev et al. [2,33]. The red border in the Figure 2 shows the enriched part of Mn, while the white border shows the poor part of Mn. The segregation degree of Fe is lower than that of Mn, the difference of element content between rich Fe region and poor Fe region is smaller, and the size of enrichment region is smaller than Mn. Element segregation also exists in the CoCrFeMnNi with 2.5 wt.% WC samples prepared by laser melting deposition. As can be seen from the EDS mapping in Figure 2d, Cr; Mn and Fe elements in the CoCrFeMnNi with 2.5 wt.% WC are separated. In the laser melting deposition process, the extremely fast heating speed brings about a great temperature gradient from the substrate to the cladding layer. In addition, due to the uneven distribution of laser radiation energy, the convection of molten pool is caused during cladding. Both may lead to composition segregation. The results of EDS point scan tests on part A and B are shown in Table 1. The CoCrFeMnNi with 2.5 wt.% WC samples have more significant component segregation, which may be due to the decomposition of WC and the formation of solid solutions.

**Figure 3:**
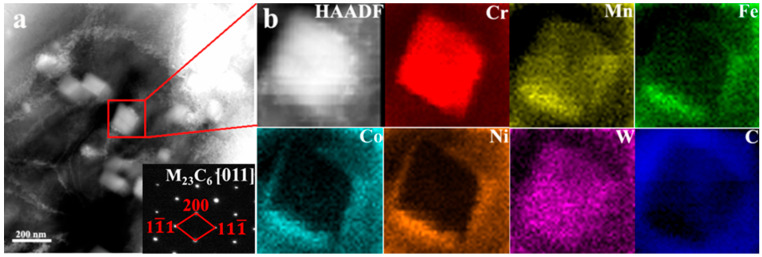
Characterization of precipitate in CoCrFeMnNi with 2.5 wt.% WC sample. (**a**) TEM image; (**b**) HAADF-STEM image and corresponding element maps.

In order to further analyze the effect of WC addition on the microstructure of the alloy, TEM and HAADF-STEM technology have been applied, as shown in Figure 3. According to Figure 3a, some nanoscale particles can be found in HEA matrix, the corresponding selected area diffraction pattern, as illustrated in Figure 3a, revealed that the precipitations were M_23_C_6_ carbides. The HAADF-STEM technology was used to characterize the particle in the red frame, the results are shown in Figure 3b. The particle is mainly composed of Cr, Mn, and W elements. This indicates that the particles in the matrix are precipitates related to the decomposition of WC and the formation of solid solutions. After the decomposition of WC, both W and C elements are solidly dissolved into the matrix, and some W elements also form nano precipitates with other elements. The EDS point scan results in Table 1 show that the alloy composition at B location contains high Cr and C elements, with a high probability of Cr carbides. However, the results of HAADF in Figure 3 show that M_23_C_6_-type carbides rich in Cr, Mn, and W appear in LMDed HEA with 2.5 wt.% WC alloy, which corresponds to the EDS results. From Figure 2(b2), it can be observed that the particle (M_23_C_6_, M is Cr, Mn, W) mainly appears at the grain boundary, this may lead to the formation of Cr poor zone along the grain boundary.

### 3.2. Electrochemical Corrosion Properties

**Figure 4:**
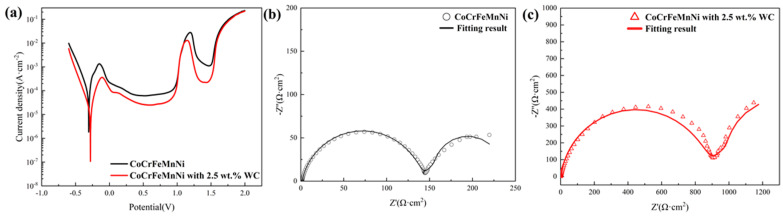
(**a**) Potentiodynamic polarization curves and electrochemical impedance spectroscopy of (**b**) CoCrFeMnNi and (**c**) CoCrFeMnNi with 2.5 wt.% WC in 0.5 M H_2_SO_4_ solution.

The potentiodynamic polarization curve test is an effective method to evaluate the corrosion behavior of materials. The polarization curves of CoCrFeMnNi and CoCrFeMnNi with 2.5 wt.% WC in 0.5 M H_2_SO_4_ are shown in Figure 4a. The characteristic parameters used to describe corrosion properties can be obtained from Figure 4a. In this work, Taffel’s extrapolation method is used to calculate the results of the polarization curves. The fitting electrochemical parameters are shown in Table 2. The corrosion potential (E_coor_) represents the corrosion potential of a material in the open circuit condition, and I_corr_ represents the corrosion current density. It is clear from both the fitting parameters and the potentiodynamic polarization curves of the alloys that CoCrFeMnNi with 2.5 wt.% WC has higher E_corr_ and lower I_corr_, which represent higher corrosion resistance. As can be seen from Figure 4a, both samples exhibit strong “activation-passivation” behavior, with wide primary passivation intervals and secondary passivation phenomena. In order to investigate the passivation process, AC electrochemical impedance tests were carried out, and the results were shown in the form of Nyquist diagram in Figure 4b. The Nyquist plots of samples are semicircular capacitance arcs. Generally, the larger the curvature radius of the arc, the stronger the corrosion resistance. It can be seen from the figure that the semi-arc of CoCrFeMnNi with 2.5 wt.% WC has a larger curvature radius, indicating its better corrosion resistance, which is consistent with the results of the polarization curve.

**Figure 5:**
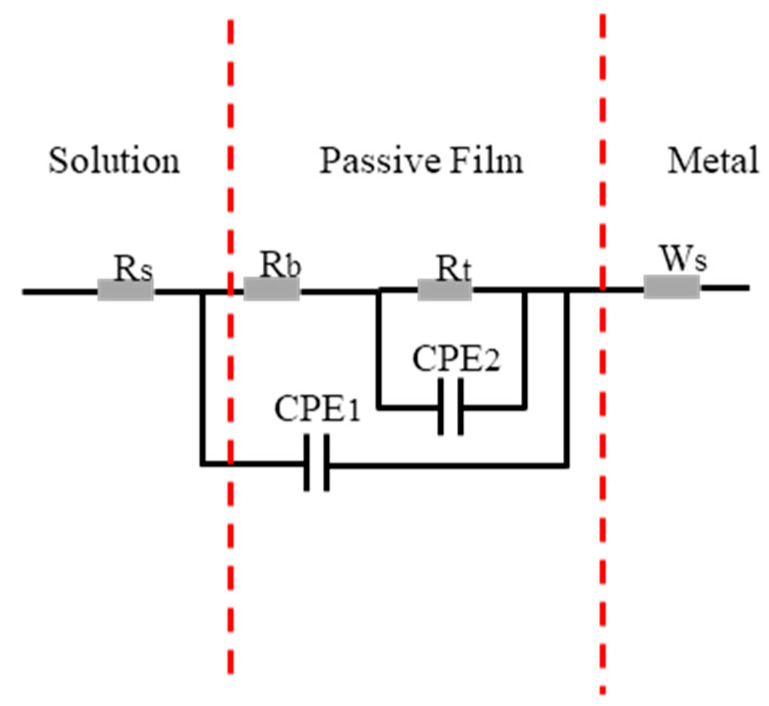
AC impedance spectrum fitting circuit diagram of CoCrFeMnNi and CoCrFeMnNi with 2.5 wt.% WC.

As Warburg impedance in the low-frequency part of Nyquist diagram appears, R(Q(R(QR)))(W) models are used to fit the results, and the equivalent electrical circuit diagram is shown in Figure 5. The fitting results are shown in Table 3, where R_s_ stands for solution resistance, R_b_ stands for film resistance, R_t_ stands for charge transfer resistance, and W_s_ represents the Warburg diffusion impedance, which is a very slow process. Before it affects the corrosion of the alloy, the alloy undergoes severe corrosion due to other reasons, and the corrosion resistance of the alloy is generally not determined by W_s_. Therefore, in AC impedance testing, the W_s_ results are generally not discussed, constant phase element CPE stands for non-ideal capacitance caused by the non-uniform electrode, and its impedance value is given by the following formula:

$$Z=\frac{1}{T{\left(j\omega \right)}^{n}}$$

*T* is the scale factor, *j* is the imaginary number unit, *ω* is the angular frequency, and *n* is the phase shift, which is between 0 and 1. When *n* = 0, CPE behaves as a pure resistance. When *n* = 1, CPE is equivalent to a pure capacitor [34]. Generally, when the chi-square value is between 10^−3^ and 10^−4^, it indicates that the fitting results are reliable. In this paper, the chi-square values of all the results are between 10^−3^ and 10^−4^. R_t_ value is positively correlated with corrosion resistance. The larger the R_t_ value, the more difficult the charge transfer and the better the corrosion resistance. It can be seen from Table 3 that the R_t_ value of CoCrFeMnNi with 2.5 wt.% WC sample is 859.1 Ω·cm^2^, about six-fold that of CoCrFeMnNi sample.

**Table 3:** Equivalent circuit fitting parameters of CoCrFeMnNi and CoCrFeMnNi with 2.5 wt.% WC in 0.5 M H_2_SO_4_ solution.

	CoCrFeMnNi	CoCrFeMnNi with 2.5 wt.% WC
R_s_ (Ω·cm^2^)	1.143	0.460
R_b_ (Ω·cm^2^)	0.411	0.806
R_t_ (Ω·cm^2^)	138.8	859.1
T_1_ (F·cm^−2^)	4.1042 × 10^−6^	2.179 × 10^−6^
n_1_	0.9304	0.986
T_2_ (F·cm^−2^)	4.442 × 10^−5^	4.081 × 10^−5^
n_2_	0.8723	0.928
Chi-square	0.829 × 10^−3^	7.199 × 10^−3^

### 3.3. Microstructure after Corrosion

**Figure 6:**
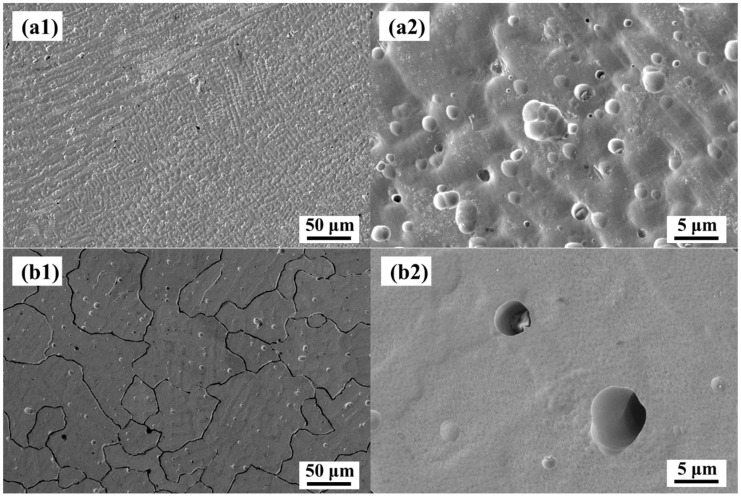
Surface morphology after dynamic potential polarization curve test in 0.5 M H_2_SO_4_ solution. (**a1**) CoCrFeMnNi; (**a2**) partial enlargement view of a1, (**b1**) CoCrFeMnNi with 2.5 wt.% WC, (**b2**) partial enlargement view of b1.

The morphology of CoCrFeMnNi and CoCrFeMnNi with 2.5 wt.% WC after the potentiodynamic polarization curve test in 0.5 M H_2_SO_4_ solution is shown in Figure 6. As can be seen in Figure 6(a1), strong corrosion occurred on the surface of the CoCrFeMnNi sample, and the dendrite structure was clearly visible. This was mainly due to the segregation of Mn and Fe elements during slow solidification, which resulted in the difference in composition between the first-solidification and post-solidification regions, and corrosion galvanic cells were formed under applied voltage. However, from the SEM images of Figure 6(a2), only a slight surface relief and partial corrosion pits were observed, indicating a large area of uniform corrosion. On the other hand, CoCrFeMnNi with 2.5 wt.% WC sample underwent relatively large corrosion along the grain boundary, which is because the C atom generated during the decomposition of WC tended to produce nanometer carbide with Cr element at the grain boundary [19,35], resulting in the formation of Cr-poor zone at the grain boundary, resulting in relatively severe corrosion at the grain boundary [36], as can be seen in Figure 6(b1).

**Figure 7:**
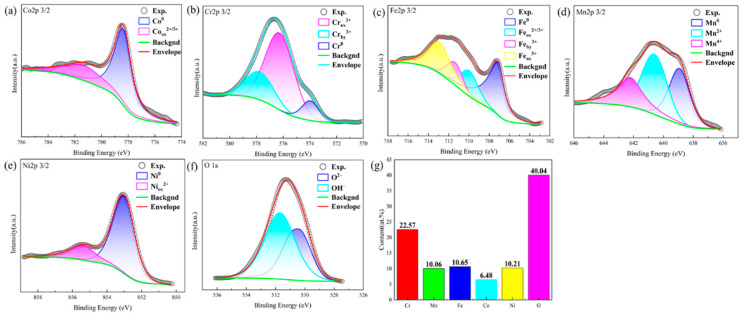
High resolution XPS spectra of the passive films formed on the CoCrFeMnNi after dynamic potential polarization curve test in 0.5 M H_2_SO_4_ solution (**a**) Co, (**b**) Cr, (**c**) Fe, (**d**) Mn, (**e**) Ni, (**f**) O, (**g**) elemental fractions in the passive film of HEA obtained by XPS analysis.

**Figure 8:**
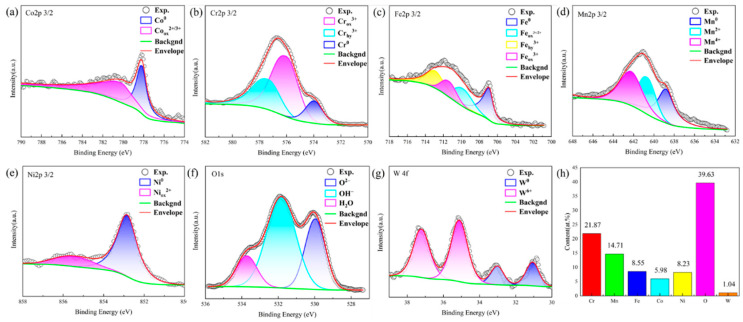
High resolution XPS spectra of the passive films formed on the CoCrFeMnNi with 2.5 wt.% WC after dynamic potential polarization curve test in 0.5 M H_2_SO_4_ solution (**a**) Co, (**b**) Cr, (**c**) Fe, (**d**) Mn, (**e**) Ni, (**f**) O, (**g**) W; (**h**) elemental fractions in the passive film of HEA obtained by XPS analysis.

To elucidate the improvement of corrosion resistance of CoCrFeMnNi with 2.5 wt.% WC alloy, XPS was carried out to study the composition and valence state of the passivation film formed after the corrosion. The high-resolution spectra of O 1 s, Fe 2p3/2, Cr 2p3/2, Ni 2p3/2, Co 2p3/2, Mn 2p3/2, and W 4f are shown in Figure 7a–f and Figure 8a–g, and the element distribution in the passivated film is shown in Figure 7g and Figure 8h. The spectrum of Co 2p3/2 in the passivated film is composed of Co^0^ and Co_ox_^3+/2+^; Co_ox_^3+/2+^ is related to its oxides CoO and Co_3_O_4_. Although CoO is easily dissolved in acidic solution, it may be formed in air, and oxide can also be formed in the anode polarization process [37,38]. The spectrum of Cr 2p3/2 consists of Cr^0^, Cr_ox_^3+^ and Cr_hy_^3+^, among which Cr_ox_^3+^ is related to Cr_2_O_3_, FeCr_2_O_4_ or NiCr_2_O_4_, while Cr_hy_^3+^ is related to Cr(OH)_3_ [39,40,41], in which Cr_ox_^3+^ accounts for 53.08%. The proportion of Cr_hy_^3+^ is 33.47%, indicating that the main valence state of Cr element in the passivation film is Cr_ox_^3+^. While Cr_2_O_3_ and Cr(OH)_3_ are considered to be the key point to the quality of the passivation film [42], Cr_2_O_3_ and Cr(OH)_3_ accounting for 86.55% in total, and Cr element accounts for 21.87% in the whole passivation film, which is much bigger than other elements. The spectrum of Fe 2p3/2 is complicated, and there are many possible substances with overlapping binding energy, which is very difficult to distinguish. As shown in Figure 7c and Figure 8c, the spectrum is divided into constituent peaks representing Fe, Fe_ox_^2+/3+^, Fe_ox_^3+^, and Fe_hy_^3+^. After anode polarization in 0.5 M H_2_SO_4_ solution, FeO is difficult to exist [43] and is not easy to form in dry air; therefore, the compounds related to Fe_ox_^2+/3+^ may be Fe_3_O_4_ and FeCr_2_O_4_. Fe_ox_^3+^ is related to Fe_2_O_3_, and due to the spectral overlap of Fe_2_O_3_ and its complex composition, NiFe_2_O_4_ may be related with Fe_ox_^3+^. Fe_hy_^3+^ comes from Fe(OH)_3_ or FeOOH [37,38,39,40]. From the figure, the relative intensities and the peak areas of Fe_ox_^2+/3+^, Fe_ox_^3+^, and Fe_hy_^3+^ are similar. The Mn 2p3/2 spectrum consists of Mn^0^, Mn^2+^, Mn^3+^ and Mn^4+^. Mn^2+^ is connected with MnO; Mn^3+^ may be Mn_2_O_3_ or MnOOH, and Mn^4+^ is related to MnO_2_. The spectrum of Ni 2p3/2 consists of Ni^0^ and Ni_ox_^2+^. Ni_ox_^2+^ is related to NiO, NiFe_2_O_4_, or NiCr_2_O_4_ [37,44]. Comparing the peak intensity, it is found that the valence state of Ni is mainly Ni^0^. As shown in Figure 8f, unlike the CrMnFeCoNi alloy, O1s spectrum of the CoCrFeMnNi with 2.5 wt.% WC alloy is composed of O^2−^, OH^−^, and H_2_O, which correspond to metal oxides and hydroxides in the passivation film. H_2_O may be the binding water formed in the passivation film [45,46]; Luo et al. reported the same results—that the binding water can be an effective substance to capture dissolved metal ions, and a new film forms to resist further corrosion [47]. It can be found, by analyzing the peak intensity, that O in the passivated film comes from a large number of metal hydroxides, which corresponds to the spectrum of other elements. The spectra of W 4f are mainly W^0^ and W^6+^, and W^6+^ is related to WO_3_ [48,49]. 

In summary, the valence states of each element in CoCrFeMnNi and CoCrFeMnNi with 2.5 wt.% WC are similar, except for oxygen element. Only CoCrFeMnNi with 2.5 wt.% WC alloy is composed of H_2_O. The corrosion resistance of the metal is highly dependent on the composition and structure of the passivation film formed in the solution, among which Cr is considered to be the main reason for the corrosion resistance of stainless steel. As can be seen from the element distribution in Figure 8h, the passivation film is mainly composed of the oxides/hydroxides of Cr(Cr_2_O_3_, Cr(OH)_3_), whose content reaches 21.87% equivalent to 304 L stainless steel. The content of Mn in the passivated film hardly decreases compared with the nominal composition of the alloy, which is consistent with the literature [50]. Carbon has an impact on the formation of bound water in the passivation film. If there is bound water in the passivation film, it will have a significant impact on the stability of the passivation film. The existence of bound water in the passivation film has a strong self-healing ability on the passivation film, and the bound water in the film will capture dissolved metal ions, and the new film will form to prevent further corrosion [46,47]. Adding a small amount of carbon to FeCoCrNiMn will increase the content of bound water in the passivation film, so that the corrosion resistance of the passivation film is improved. In the passivation film formed by the CoCrFeMnNi with 2.5 wt.% WC in 0.5 M H_2_SO_4_ solution, the content of bound water is 4.49%, which will improve the corrosion resistance of the passivation film. In addition, the addition of W can inhibit the dissolution of metal in acidic electrolyte and also improve the corrosion resistance of the alloy.

## 4. Conclusions

In this study, LMDed CoCrFeMnNi with 2.5 wt.% WC HEA particle were fabricated, and a comparative study on the electrochemical corrosion behavior of the CoCrFeMnNi and CoCrFeMnNi with 2.5 wt.% WC particle in 0.5 M H_2_SO_4_ was carried out. The influence of WC on the corrosion resistance of CoCrFeMnNi was investigated, and the conclusions are as follows:

(1) The microstructure of CoCrFeMnNi with WC particle prepared by laser melting deposition is composed of columnar crystals and equiaxed crystals. During the preparation process, WC particles were decomposed, and elements C and W were incorporated into the CoCrFeMnNi matrix, resulting in strong lattice distortion;
(2) The electrochemical measurement results show that CoCrFeMnNi with 2.5 wt.% WC have a smaller corrosion current density of 1.594 × 10^−5^ A·cm^−2^ and larger corrosion potential −0.285 V_Ag/AgCl_ and higher charge transfer 859.1 Ω·cm^2^, showing better corrosion resistance than CoCrFeMnNi;
(3) The morphology after corrosion shows that the CoCrFeMnNi has a large area of uniform corrosion, while the CoCrFeMnNi with 2.5 wt.% WC corrodes along the grain boundary; furthermore, the XPS results of the passive film show that the content of Cr_2_O_3_ and Cr(OH)_3_ are high, which is helpful to improve the stability of the passive film, and additionally, that the decomposition of WC is not a bad thing. The incorporation of C atoms causes the combined water to appear in the passive film, which makes the passive film have a self-repairing function and improves its corrosion resistance. In addition, the addition of W can inhibit the dissolution of metal in acidic electrolyte and also improve the corrosion resistance of the alloy.

## Figures and Tables

**Table 1:** Composition of CoCrFeMnNi and CoCrFeMnNi with 2.5 wt.% WC high-entropy alloys A and B regions in Figure 2(a2,b2).

		Cr	Mn	Fe	Co	Ni	C	W
LMDed HEA	A	20.40	23.45	20.64	18.26	17.25	—	—
	B	23.87	19.94	20.55	18.37	17.27	—	—
LMDed HEA with 2.5 wt.% WC	A	21.59	21.55	19.07	18.00	18.94	0.55	0.30
	B	26.68	19.07	15.00	16.20	15.65	6.87	0.53

**Table 2:** Electrochemical corrosion parameters of CoCrFeMnNi and CoCrFeMnNi with 2.5 wt.% WC obtained from potentiodynamic polarization curve measured in 0.5 M H_2_SO_4_ solution.

Sample	CoCrFeMnNi	CoCrFeMnNi with 2.5 wt.% WC
E_corr_(V_Ag/AgCl_)	−0.298	−0.285
i_corr_(A/cm^−2^)	3.038 × 10^−5^	1.594 × 10^−5^
E_pp_(V_Ag/AgCl_)	−0.14	−0.107
i_pass_(A/cm^2^)	6.275 × 10^−5^	2.519 × 10^−5^
E_b_(V_Ag/AgCl_)	0.961	0.926
E_sp_(V_Ag/AgCl_)	1.196	1.147
ΔE(V_Ag/AgCl_)	1.101	1.033

E_pp_: primary passivation potential i_pass_: Passivation current density E_b_: breakdown potential. E_sp_: secondary passivation potential ΔE: E_b_ − E_pp_, length of passive zone.

## Data Availability

The data presented in this work are available on request from the corresponding authors.

## References

[1] Yeh J.W., Chen S.K., Lin S.J., Gan J.Y., Chin T.S., Shun T.T., Tsau C.H., Chang S.Y. (2004). Nanostructured high-entropy alloys with multiple principal elements: Novel alloy design concepts and outcomes. Adv. Eng. Mater..

[2] Cantor B., Chang I.T.H., Knight P.C., Vincent A. (2004). Microstructural development in equiatomic multicomponent alloys. Mater. Sci. Eng. A.

[3] Miracle D.B., Senkov O.N. (2017). A critical review of high entropy alloys and related concepts. Acta Mater..

[4] Otto F., Dlouhý A., Somsen C., Bei H., Eggeler G., George E.P. (2013). The influences of temperature and microstructure on the tensile properties of a CoCrFeMnNi high-entropy alloy. Acta Mater..

[5] Luan H., Zhang X., Ding H., Zhang F., Luan J., Jiao Z., Yang Y.C., Bu H., Wang R., Gu J. (2022). High-entropy induced a glass-to-glass transition in a metallic glass. Nat. Commun..

[6] Peng Z., Sun J., Luan H.W., Chen N., Yao K.F. (2023). Effect of Mo on the high temperature oxidation behavior of Al_19_Fe_20-x_Co_20-x_Ni_41_Mo_2x_ high entropy alloys. Intermetallics.

[7] Lei Z.F., Liu X.J., Wu Y., Wang H., Jiang S., Wang S.D., Hui X.D., Wu Y.D., Gault B., Kontis P. (2018). Enhanced strength and ductility in a high-entropy alloy via ordered oxygen complexes. Nature.

[8] Lu Z.P., Wang H., Chen M.W., Baker I., Yeh J.W., Liu C.T., Nieh T.G. (2015). An assessment on the future development of high-entropy alloys: Summary from a recent workshop. Intermetallics.

[9] Zhang Y., Zuo T.T., Tang Z., Gao M.C., Dahmen K.A., Liaw P.K., Lu Z.P. (2014). Microstructures and properties of high-entropy alloys. Prog. Mater Sci..

[10] Peng Z., Li B.W., Luo Z.B., Chen X.F., Tang Y., Yang G.N., Gong P. (2023). A Lightweight AlCrTiV_0.5_Cu_x_ High-Entropy Alloy with Excellent Corrosion Resistance. Materials.

[11] Chen X.F., Wang Q., Cheng Z.Y., Zhu M.L., Zhou H., Jiang P., Zhou L.L., Xue Q., Yuan F.P., Zhu J. (2021). Direct observation of chemical short-range order in a medium-entropy alloy. Nature.

[12] George E.P., Raabe D., Ritchie R.O. (2019). High-entropy alloys. Nat. Rev. Mater..

[13] Peng Z., Luo Z.B., Li B.W., Li J.F., Luan H.W., Gu J.L., Wu Y., Yao K.F. (2022). Microstructure and mechanical properties of lightweight AlCrTiV_0.5_Cu_x_ high-entropy alloys. Rare Met..

[14] Jia Y.F., Jia Y.D., Wu S.W., Ma X.D., Wang G. (2019). Novel Ultralight-Weight Complex Concentrated Alloys with High Strength. Materials.

[15] Senkov O.N., Senková S., Miracle D.B., Woodward C. (2013). Mechanical properties of low-density, refractory multi-principal element alloys of the Cr–Nb–Ti–V–Zr system. Mater. Sci. Eng. A.

[16] Ren J., Zhang Y., Zhao D.X., Chen Y., Guan S., Liu Y.F., Liu L., Peng S.Y., Kong F., Poplawsky J.D. (2022). Strong yet ductile nanolamellar high-entropy alloys by additive manufacturing. Nature.

[17] Xiang S., Luan H.W., Wu J., Yao K.F., Li J.F., Liu X., Tian Y.Z., Mao W.L., Bai H., Le G.M. (2019). Microstructures and mechanical properties of CrMnFeCoNi high entropy alloys fabricated using laser metal deposition technique. J. Alloys Compd..

[18] Xiang S., Li J., Luan H.W., Amar A., Lu S.Y., Li K., Zhang L., Liu X., Le G.M., Wang X.Y. (2019). Effects of process parameters on microstructures and tensile properties of laser melting deposited CrMnFeCoNi high entropy alloys. Mater. Sci. Eng. A.

[19] Li J.F., Xiang S., Luan H.W., Amar A., Liu X., Lu S.Y., Zeng Y.Y., Le G.M., Wang X.Y., Qu F.S. (2019). Additive manufacturing of high-strength CrMnFeCoNi high-entropy alloys-based composites with WC addition. J. Mater. Sci. Technol..

[20] Chew Y., Bi G.J., Zhu Z.G., Ng F.L., Weng F., Liu S.B., Nai S.M.L., Lee B.Y. (2019). Microstructure and enhanced strength of laser aided additive manufactured CoCrFeNiMn high entropy alloy. Mater. Sci. Eng. A.

[21] Wang Y.M., Voisin T., McKeown J.T., Ye J., Calta N.P., Li Z., Zeng Z., Zhang Y., Chen W., Roehling T.T. (2018). Additively manufactured hierarchical stainless steels with high strength and ductility. Nat. Mater..

[22] Melia M.A., Carroll J.D., Whetten S.R., Esmaeely S.N., Locke J., White E., Anderson I., Chandross M., Michael J.R., Argibay N. (2019). Mechanical and Corrosion Properties of Additively Manufactured CoCrFeMnNi High Entropy Alloy. Addit. Manuf..

[23] Jia Y.J., Chen H.N., Liang X.D. (2019). Microstructure and wear resistance of CoCrNbNiW high-entropy alloy coating prepared by laser melting deposition. Rare Met..

[24] Moghaddam A.O., Shaburova N.A., Samodurova M.N., Abdollahzadeh A., Trofimov E.A. (2021). Additive manufacturing of high entropy alloys: A practical review. J. Mater. Sci. Technol..

[25] Sun Z., Tan X.P., Descoins M., Mangelinck D., Tor S.B., Lim C.S. (2019). Revealing hot tearing mechanism for an additively manufactured high-entropy alloy via selective laser melting. Scr. Mater..

[26] Guo J., Liu C.H., Wang D.X., Xu L.F., Song K.K., Gao M. (2023). Structure and Wear Resistance of TiC-Reinforced Al1.8CrCuFeNi2 High-Entropy Alloy Coating Using Laser Cladding. Materials.

[27] Chen P., Li S., Zhou Y.H., Yan M., Attallah M.M. (2020). Fabricating CoCrFeMnNi high entropy alloy via selective laser melting in-situ alloying. J. Mater. Sci. Technol..

[28] Kim Y.K., Cho J., Lee K.A. (2019). Selective laser melted equiatomic CoCrFeMnNi high-entropy alloy: Microstructure, anisotropic mechanical response, and multiple strengthening mechanism. J. Alloys Compd..

[29] Zhang Z.J., Mao M.M., Wang J.G., Gludovatz B., Zhang Z., Mao S.X., George E.P., Yu Q., Ritchie R.O. (2015). Nanoscale origins of the damage tolerance of the high-entropy alloy CrMnFeCoNi. Nat. Commun..

[30] Xu Z.L., Zhang H., Du X.J., He Y.Z., Luo H., Song G.S., Mao L., Zhou T.W., Wang L.L. (2020). Corrosion resistance enhancement of CoCrFeMnNi high-entropy alloy fabricated by additive manufacturing. Corros. Sci..

[31] Yehia H.M. (2019). Microstructure, physical, and mechanical properties of the Cu/ (WC-TiC-Co) nano-composites by the electroless coating and powder metallurgy technique. J. Compos. Mater..

[32] Hassan M.A., Yehia H.M., Mohamed A.S.A., El-Nikhaily A.E., Elkady O.A. (2021). Effect of Copper Addition on the AlCoCrFeNi High Entropy Alloys Properties via the Electroless Plating and Powder Metallurgy Technique. Crystals.

[33] Salishchev G., Tikhonovsky M.A., Shaysultanov D., Stepanov N.D., Kuznetsov A.V., Kolodiy I.V., Tortika A.S., Senkov O.N. (2014). Effect of Mn and V on structure and mechanical properties of high-entropy alloys based on CoCrFeNi system. J. Alloys Compd..

[34] Kissi M., Bouklah M., Hammouti B., Benkaddour M. (2006). Establishment of equivalent circuits from electrochemical impedance spectroscopy study of corrosion inhibition of steel by pyrazine in sulphuric acidic solution. Appl. Surf. Sci..

[35] Park J.M., Choe J., Kim J.G., Bae J.W., Moon J., Yang S., Kim K.T., Yu J.H., Kim H.S. (2019). Superior tensile properties of 1%C-CoCrFeMnNi high-entropy alloy additively manufactured by selective laser melting. Mater. Res. Lett..

[36] Samoilova O., Pratskova S., Shaburova N., Ostovari Moghaddam A., Trofimov E. (2023). Corrosion Resistance of Al_0.5_CoCrFeNiCu_x_Ag_y_ (x = 0.25, 0.5; y = 0, 0.1) High-Entropy Alloys in 0.5M H_2_SO_4_ Solution. Materials.

[37] Luo H., Li Z.M., Mingers A.M., Raabe D. (2018). Corrosion behavior of an equiatomic CoCrFeMnNi high-entropy alloy compared with 304 stainless steel in sulfuric acid solution. Corros. Sci..

[38] Gardin E., Zanna S., Seyeux A., Allion-Maurer A., Marcus P. (2019). XPS and ToF-SIMS characterization of the surface oxides on lean duplex stainless steel – Global and local approaches. Corros. Sci..

[39] Luo H., Zou S.W., Chen Y.H., Li Z., Du C.W., Li X.G. (2020). Influence of carbon on the corrosion behaviour of interstitial equiatomic CoCrFeMnNi high-entropy alloys in a chlorinated concrete solution. Corros. Sci..

[40] Sun Y.P., Wang Z., Yang H.J., Lan A.D., Qiao J.W. (2020). Effects of the element La on the corrosion properties of CrMnFeNi high entropy alloys. J. Alloys Compd..

[41] Pourbaix M. (1974). Atlas of Electrochemical Equilibria in Aqueous Solutions.

[42] Haupt S., Strehblow H.H. (1995). Combined Surface Analytical and Electrochemical Study of the Formation of Passive Layers on Fe/Cr Alloys in 0.5 M H_2_SO_4_. ChemInform.

[43] Mansour C., Lefèvre G., Pavageau E.M., Catalette H., Fédoroff M., Zanna S. (2009). Sorption of sulfate ions onto magnetite. J. Colloid Interface Sci..

[44] Okamoto G.O., Shibata T. (1965). Desorption of Tritiated Bound-water from the Passive Film Formed on Stainless Steels. Nature.

[45] Jiang Y., Yang J.F., Liu R., Wang X.P., Fang Q.F. (2014). Oxidation and corrosion resistance of WC coated tungsten fabricated by SPS carburization. J. Nucl. Mater..

[46] Noji N., Kashiwagura K., Akao N., Soma S., Hara N., Sugimoto K. (2002). Corrosion resistance of tungsten and tungsten alloys for spallation target in stagnant and flowing water. J. Jpn. Inst. Met..

[47] Hsu K.M., Chen S.H., Lin C.S. (2021). Microstructure and corrosion behavior of FeCrNiCoMn_x_ (x = 1.0, 0.6, 0.3, 0) high entropy alloys in 0.5 M H_2_SO_4_. Corros. Sci..

[48] Wang C.M., Yu Y., Zhang H., Xu L.X., Ma X.Y., Wang F.F., Song B.Y. (2021). Microstructure and corrosion properties of laser remelted CrFeCoNi and CrMnFeCoNi high entropy alloys coatings. J. Mater. Res. Technol..

[49] Zhao Q.H., Liu W., Li S.Z., Zhang B.L., Zhu Y.C., Lu M.X. (2016). Effects of W and Mo Additions on Wet-dry Acid Corrosion Behavior of Low-Alloy Steels Under Different O_2_ Concentrations. Acta Metall. Sin. (Engl. Lett.).

[50] Ma M.Y., He C.L., Chen L.Q., Wei L.L., Misra R.D.K. (2018). Effect of W and Ce additions on the electrochemical corrosion behaviour of 444-type ferritic stainless steel. Corros. Eng. Sci. Technol..

